# *Entamoeba histolytica*: Proteomics Bioinformatics Reveal Predictive Functions and Protein–Protein Interactions of Differentially Abundant Membrane and Cytosolic Proteins

**DOI:** 10.3390/membranes11060376

**Published:** 2021-05-21

**Authors:** Norhidayah Azmi, Nurulhasanah Othman

**Affiliations:** Institute for Research in Molecular Medicine (INFORMM), Universiti Sains Malaysia, Pulau Pinang 11800, Malaysia; hidayah84@student.usm.my

**Keywords:** *Entamoeba histolytica*, membrane protein, cytosolic protein, differential protein abundance, functional annotation, protein–protein interaction

## Abstract

Amoebiasis is caused by *Entamoeba histolytica* and ranked second for parasitic diseases causing death after malaria. *E.* *histolytica* membrane and cytosolic proteins play important roles in the pathogenesis. Our previous study had shown several cytosolic proteins were found in the membrane fraction. Therefore, this study aimed to quantify the differential abundance of membrane and cytosolic proteins in membrane versus cytosolic fractions and analyze their predicted functions and interaction. Previous LC-ESI-MS/MS data were analyzed by PERSEUS software for the differentially abundant proteins, then they were classified into their functional annotations and the protein networks were summarized using PantherDB and STRiNG, respectively. The results showed 24 (44.4%) out of the 54 proteins that increased in abundance were membrane proteins and 30 were cytosolic proteins. Meanwhile, 45 cytosolic proteins were found to decrease in abundance. Functional analysis showed differential abundance proteins involved in the molecular function, biological process, and cellular component with 18.88%, 33.04% and, 48.07%, respectively. The STRiNG server predicted that the decreased abundance proteins had more protein–protein network interactions compared to increased abundance proteins. Overall, this study has confirmed the presence of the differentially abundant membrane and cytosolic proteins and provided the predictive functions and interactions between them.

## 1. Introduction

Amoebiasis or amoebic dysentery is a protozoan disease caused by *Entamoeba histolytica*, which is mainly found in the human colon. It may exist as a non-pathogenic commensal or penetrate the intestinal mucosa and metastasize to cause an extraintestinal infection like an amoebic liver abscess (ALA) [1]. The parasite has a simple two-stage life cycle including trophozoite and cyst stages. Infection of a human by *E. histolytica* begins by ingestion of the cyst, which is protected from the environment by a highly resistant chitin-containing cell wall [2]. Trophozoites penetrate the intestinal mucus layer that develops colitis in the colon during the disease-causing process. Trophozoite invasion includes the destruction of epithelial cells, lymphocytes, and polymorphonuclear cells [2]. 

Many studies have been performed on the trophozoites form because it is easy to cultivate. During the infection, surface molecules of trophozoites connect the host to the parasite, which is crucial in tissue invasion, colitis induction, and the development of liver abscess [3]. However, little is understood about the *E. histolytica* membrane molecules. Membrane proteins can be found on the cell surface, reticulum endoplasm (ER), Golgi apparatus, endomembrane, mitochondria and nucleus. In *E. histolytica*, host–parasite interactions occur on the membrane surface, which is the outer layer of the trophozoites, and the adherence process is initiated by Gal/GalNAc lectin membrane protein, which is the first step in developing the disease. This adherence is important in mediating killing and/or phagocytosis and exposing the surface molecules to the host’s immune system [2]. 

Membrane proteins possess transmembrane (TM) domains, which differentiate them from cytosolic proteins [4]. They are responsible for cellular adhesion and recognition, molecular receptors, substrate transportation through membranes, signal transduction, protein secretion, enzymatic activity [5] and/or signal peptides, which assists protein positioning in cellular organelles or the cell membrane [6]. To date, only around 20 proteins or protein families of *E. histolytica* membrane have been identified [3]. Therefore, it is important to further identify *E. histolytica* membrane proteins since a majority of the protein functions are unexplored.

Critical steps for protein analysis in a biological context are extraction and isolation of proteins from chemical and physical interactions with other biomolecules from specific cells [7]. Hence, our previous extensive study (Ujang et al., (2018) [7]) performed three different extraction methods to fractionate membrane and cytosolic proteins of *E. histolytica* trophozoites. The extractions were performed to isolate the total membrane and not for an organelle specific-membrane fraction. The membrane and cytosolic fractions were analyzed by LC-ESI-MS/MS for protein identification to indicate the presence or absence of the membrane and cytosolic proteins in the fractions. The result showed a commercial kit, ProteoPrep (Sigma-Aldrich, Darmstadt, Germany) was the best method for the membrane protein extraction in terms of its sensitivity and specificity by analyzing membrane and cytosolic fractions. Mixed membrane and cytosolic proteins accounted for 267, 127 and 130 identified proteins in both fractions [7]. The cytosolic proteins were found to be present in the membrane fraction from the three extraction methods. This phenomenon has raised our curiosity as to whether the cytosolic proteins were really located and worked hand in hand with the membrane proteins or this phenomenon occurred because of the technique used during the membrane extraction step. 

Using the previous MS data of label-free quantification [7], we aimed to analyze the differential abundance proteins in membrane versus cytosolic fractions of *E. histolytica.* The differentially abundant proteins were analyzed using bioinformatics platforms to further understand the predictive biological functions and interactions among the proteins. In this study, the TM and signal peptide domains of identified proteins were obtained from AmoebaDB database. The database uses a specific algorithm to predict the presence of these domains in the protein. We hypothesized that the membrane proteins were increased while cytosolic proteins were decreased in abundance by comparing the membrane versus cytosolic fractions. If there were increased abundance of membrane and cytosolic proteins in the membrane fraction, we postulated there were protein–protein interactions between these two proteins. This would therefore strengthen the notion that the cytosolic proteins localized to the membrane part and functioned together with membrane proteins in *E. histolytica*.

## 2. Materials and Methods

### 2.1. PERSEUS Software Platform Analysis 

MS data of LFQ from three different extraction methods by Ujang et al., (2018) [7] were retrieved from ProteomeXchange with identifier no. PXD010171 for differential protein abundant analysis in membrane versus cytosolic fractions using PERSEUS MaxQuant software (MaxPlanck Institute of Biochemistry, Martinsried, Germany). The LFQ intensities were based on 267, 127 and 130 identified proteins in both fractions [7]. In this study, the LFQ intensities of the membrane fraction was the numerator and the cytosolic fraction was the denominator. Therefore, the differential abundance proteins presented in the result were on the proteins of the membrane fraction. The PERSEUS integrates a multitude of algorithms enabling complete analysis of MS data starting from raw LC-MS runs. Firstly, Perseus.exe was downloaded from the website www.maxquant.net/perseus/ (accessed on: 06.05.2020). The Generic Matrix Upload icon was clicked, and the icon box was selected. A new window popped out. The data frame was composed by selecting data features carefully inside the right box. The LFQ intensities of membrane and cytosolic proteins of three extraction methods were uploaded for quantification and statistical analyses along with other parameters, i.e., accession no., description of protein, score, number of peptides and coverage. Log in transformation parameter and base parameters were selected to transform the data in order to facilitate the protein abundance fold change calculation in the analysis. For data analysis, there were four different statistical tests offered by Perseus; one sample, two samples, multiple samples, and two-way ANOVA. This study used t-test for two group analysis. The q-value and False Discovery Rate (FDR) calculated were set-up to show a cut-off curve indicating which proteins were significant. The S0 (fold-change) and FDR were set to 0 and q < 0.05, respectively. Then, a volcano plot was visualized by setting the *x* and *y*-axis with log_2_ fold changes and −log_10_ *p*-values, respectively. This was done to visualize the potential or significant proteins located in the right and left of the quadrant’s plot. Finally, the statistical results were saved by exporting to a file or in .txt and it could be opened either in Excel or Perseus software. The proteins that showed differences in the fold-change (Fc) > 2 and q < 0.01 were considered significant.

### 2.2. Determination of Transmembrane and Signal Peptide Domains

The accession no. of differentially abundant proteins was searched against the AmoebaDB database release 47 (accessed on 10 June 2020). Then, information on the presence of transmembrane and signal peptide domains were obtained from the protein properties and features section.

### 2.3. Gene Ontology 

Classification of the differential abundant proteins into their molecular function, biological process, and cellular component was performed by the **P**rotein **AN**alysis **TH**rough **E**volutionary **R**elationship (PANTHER) DB version 15.0 system at www.pantherdb.org (accessed on 17 June 2020). The Gene List Analysis page was browsed, the accession number of the increased or decreased abundant proteins with fold –change (Fc) > 2 and q < 0.05 were typed in the box and the ID list was ticked. Then, *E. histolytica* was selected as the ID and functional classification was viewed in the gene list. The submit button was clicked to generate pie charts. The result was based on the analysis that was selected, i.e., Functional Classification Viewed in the Gene List. It displayed a gene list indicating mapped and unmapped genes. The system brought up the family list page if PANTHER Generic Mapping file or ID’s from Reference Proteome Genome file was selected as an input file. A multi-coloured pie chart icon was clicked to display the functional analysis results of all the proteins involved in molecular function, biological process, cellular components, and protein classes and their related pathways. Category name, and series of numbers appeared when the mouse was moved over the pie chart. The numbers represented statistics of the selected category. The legend link on the right side of the pie chart also could be used to retrieve a gene list of the category.

### 2.4. Protein–Protein Interaction Network Prediction

The www.string-db.org (version 11, accessed on 20 June 2020) webpage was navigated to analyze protein–protein interaction networks. STRiNG provides a database of known and predicted protein–protein interactions including indirect (functional) and direct (physical) associations arising from computational prediction, knowledge transferable and primary databases, such as participating in the same biological process or protein class (gene ontology). STRiNG (**S**earch **T**ool for **R**etrieval of **I**nteracting **G**enes/Proteins) can be searched by single or multiple protein names, or by amino acid sequences in any available formats. In this study, a total of 99 accession numbers derived from 54 and 45 increased and decreased abundant proteins was inserted as protein identifier. The multiple proteins input was selected. Then, the organism of interest (*E. histolytica*) was specified before clicking the SEARCH button. Then, the network was displayed to show the predicted network of association for a group of proteins. Proteins were represented by the nodes, and edges showed predicted functional associations. By clicking on a node gave several details of the protein and the edge displayed a detailed evidence breakdown. The line or edge could be seen in seven different colors, which represented different evidence used in protein relation prediction. The protein window provided information about the protein and other links related to the protein.

## 3. Results 

### 3.1. Differential Abundant Proteins 

By combining the results from mass spectrometry analysis of three extraction methods by Ujang et al., 2018 [7], a total of 99 differentially abundant proteins with 54 increased and 45 decreased abundance in membrane versus cytosolic fractions fulfilled the chosen criteria of protein fold-change (Fc) > 2 fold and q < 0.05 (Table 1). Furthermore, 24 of the increased abundant proteins were predicted as membrane proteins (Table 2) and there was no membrane protein from decreased abundance proteins (Table 3). 

### 3.2. Functional Classification of Differential Abundant Proteins.

**Figure 1:**
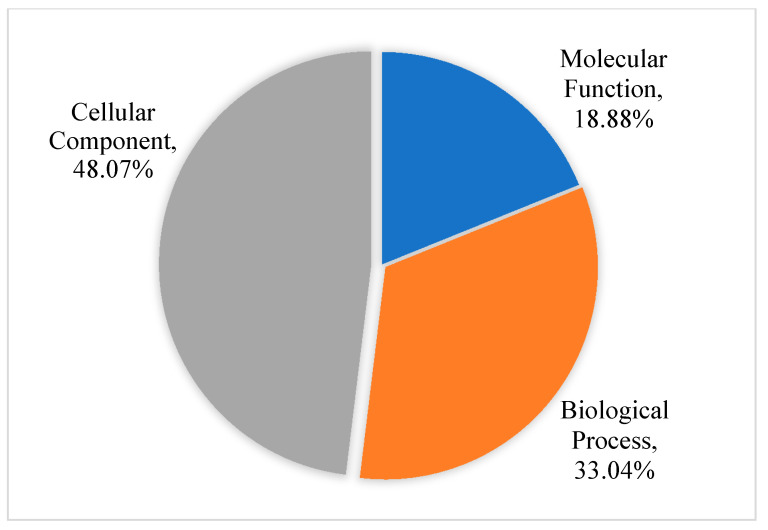
The functional analysis of increased and decreased abundance proteins in the membrane versus cytosolic fractions.

**Figure 2:**
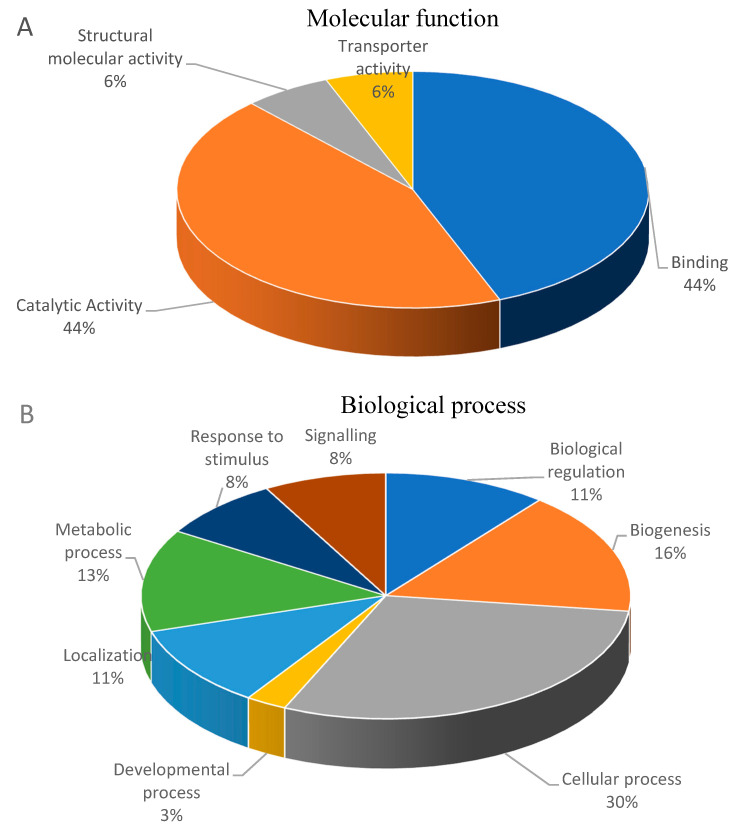

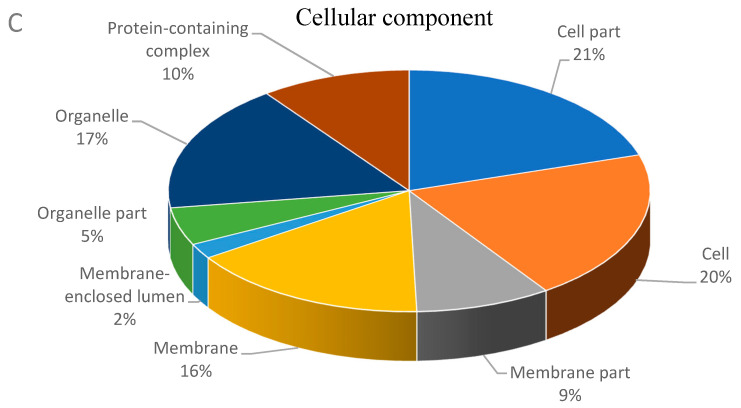
Functional classification analysis of increased abundance proteins according to their molecular function (**A**), biological process (**B**), and cellular component (**C**).

Figure 1 shows 48.07%, 33.04% and 18.88% of the differential abundant proteins involved in cellular components, biological process and molecular function, respectively. Increased abundance proteins of the membrane fraction involved in molecular function included catalytic activity (seven proteins), binding (seven proteins), structural molecular activity (one protein) and transporter activity (one protein) (Figure 2A). Figure 2B showed increased abundance proteins involved in the biological processes such as biological regulation (four proteins), biogenesis (six proteins), cellular process (eleven proteins), developmental process (one protein), localization (four proteins), metabolic process (five proteins), response to stimulus (three proteins), and signaling (three proteins). These proteins were also involved in cellular components such as cell part (twelve proteins), cell (twelve proteins), membrane part (five proteins), membrane (nine proteins), membrane-enclosed lumen (one protein), organelle part (three proteins), organelle (ten proteins), and protein-containing complex (six proteins), as shown in Figure 2C. 

**Figure 3:**
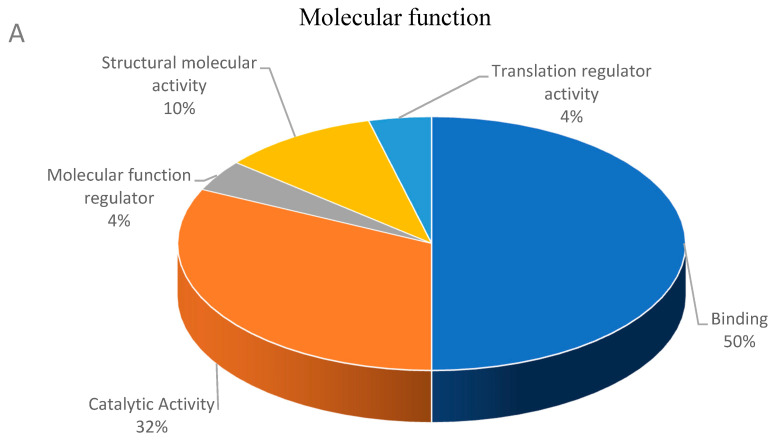

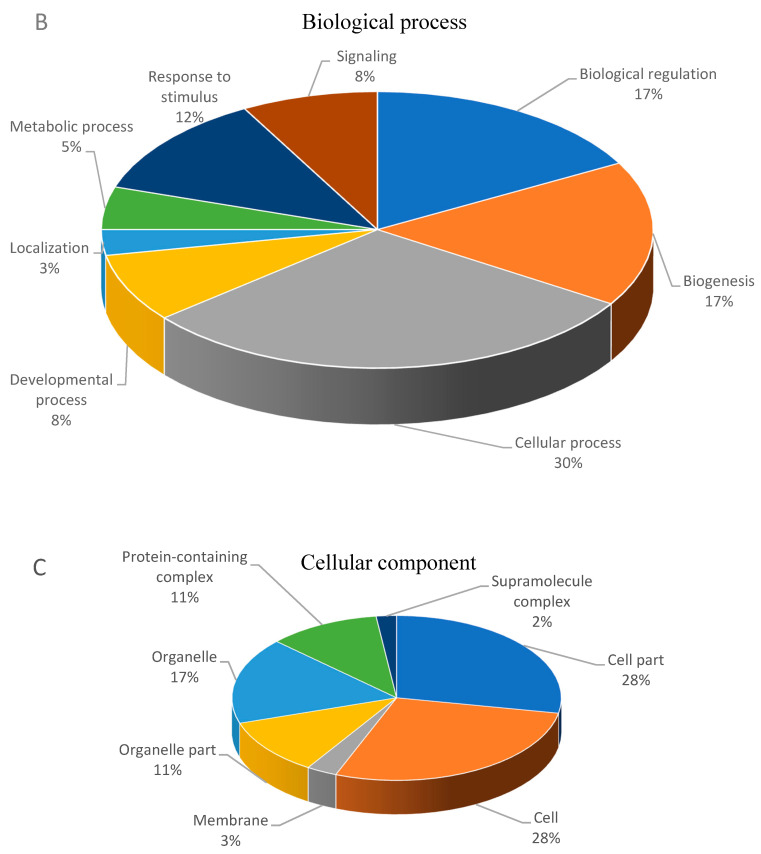
Functional classification of decreased abundance proteins according to their molecular function (**A**), biological process (**B**) and cellular component (**C**).

Decreased abundance proteins were involved in the molecular function such as binding (fourteen proteins), catalytic activity (nine proteins), molecular function regulator (one protein), structural molecular activity (three proteins), and translation regulator activity (one protein) (Figure 3A). Proteins associated with biological process involved biological regulation (seven proteins), biogenesis (seven proteins), cellular process (twelve proteins), developmental process (three proteins), localization (one protein), metabolic process (two proteins), response to stimulus (five proteins), and signaling (three proteins) (Figure 3B). These proteins were classified into cellular components such as cell and cell part (fifteen proteins each), membrane (two proteins), organelle and organelle part (nine and six proteins each), protein-containing complex (six proteins), and supramolecule complex (one protein) as shown in Figure 3C. 

Increased abundance proteins demonstrated by PANTHER DB analysis (Table S1 in Supplementary Materials) were mainly involved in binding and catalytic activity (molecular function), cellular process (biological process) and cell and cell part (cellular component). The identified proteins were Rho family GTPase protein (EHI_129750), 70 kDa heat shock protein, putative (EHI_199590), calreticulin putative (EHI_136160), 60S acidic ribosomal protein (EHI_175460), NAD(P) transhydrogenase subunit alpha putative (EHI_014030), 3-oxo 5 alpha-steroid 4-dehydrogenase domain (EHI_076870), calcium- transporting ATPase (EHI_054830), Ehsyntaxin 1A fragment (EHI_139030) and protein SEY1 homolog 2 (EHI_054180). 

On the other hand, PANTHER DB analysis showed decreased abundant proteins (Table S2 in Supplementary Materials) mainly involved in binding (molecular function), cellular process (biological process) and cell and cell part (cellular component). There were some proteins involved in the binding, cellular activities and cell/cell part such as heat shock protein 70 putative (EHI_052860), peptidyl-prolyl cis-trans isomerase (EHI_125840), elongation factor 2 (EHI_166820), filamin 2 putative (EHI_104630), actin putative (EHI_198930), Rho family GTPase (EHI_192450 and EHI_146180), profilin (EHI_176140), Skp1 family protein (EHI_174180), Rho GDP exchange inhibitor (EHI_147570), Arp2/3 complex 34 kDa subunit (EHI_091250) and F-actin-capping protein subunit beta (EHI_005020). 

### 3.3. Protein–Protein Interaction Network Prediction.

**Figure 4:**
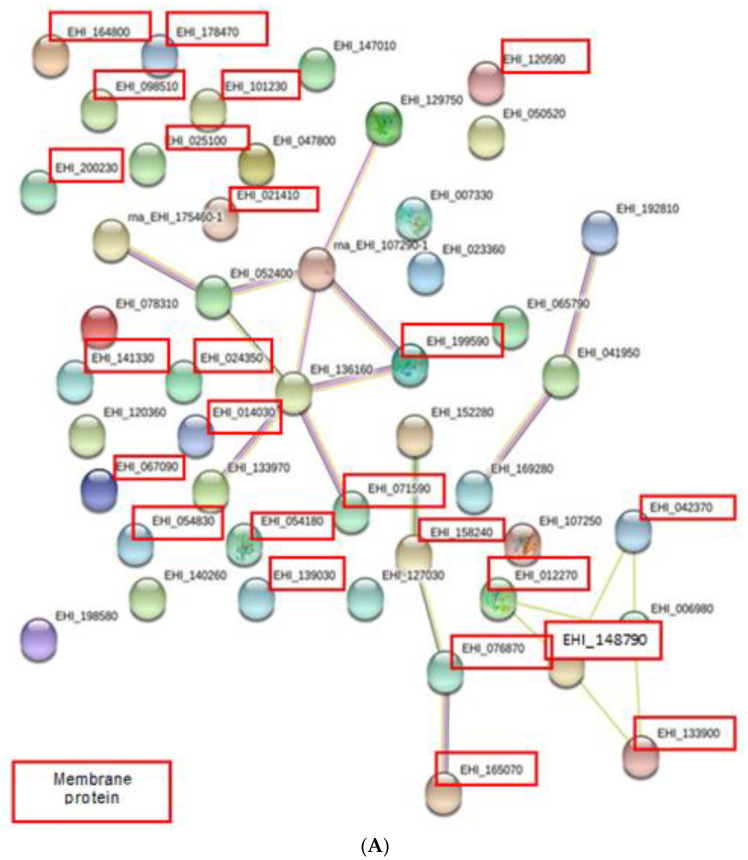

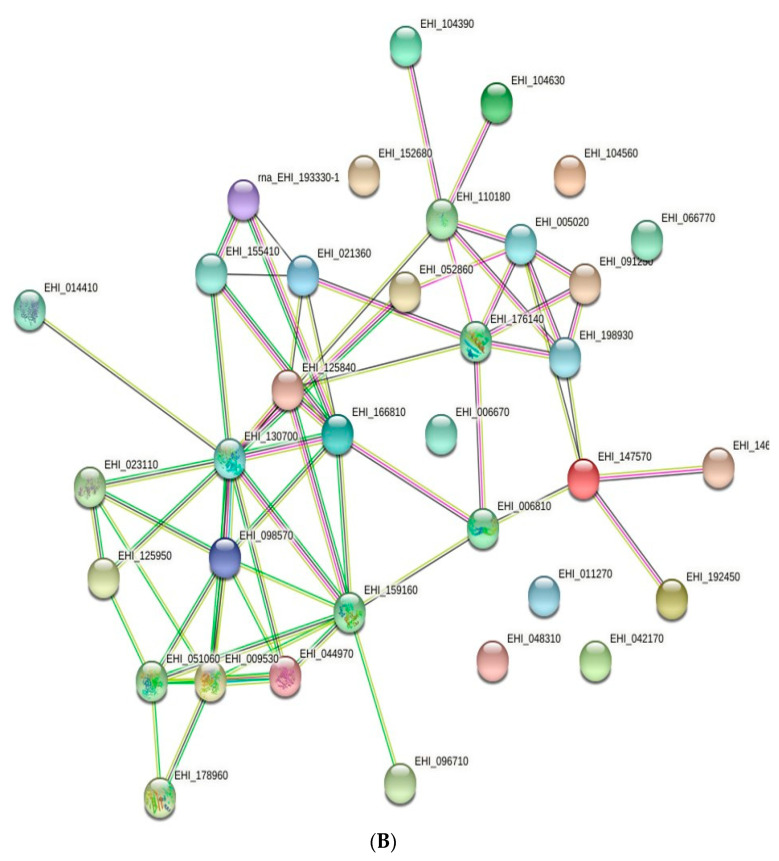
STRiNG generated networks of increased and decreased abundance proteins. (**A**) Increased abundance proteins comprised membrane and cytosolic proteins. Proteins with accession no. in the red boxes are membrane proteins. Meanwhile, proteins with accession no. without red boxes are cytosolic proteins. (**B**) Decreased abundance proteins comprised cytosolic proteins.

Highly significant interactions (*p* < 3.66 × 10^−11^) were predicted in decreased abundance proteins when compared to increased abundance proteins (*p* < 1.91 × 10^−7^) (Figure 4A,B). Analysis by STRiNG showed there were interactions between membrane and cytosolic proteins among increased abundance proteins as shown in Figure 4A. Line colors indicate types of interaction evidences (Table 4).

## 4. Discussion

In this study, there were increased and decreased abundance proteins in membrane versus cytosolic fractions from combining all three extraction methods. In this study, 24 (44.4%) out of 54 increased abundance proteins in the membrane fraction were confirmed membrane proteins as they had a transmembrane region and/or single peptide (Table 3 and Table 4). Meanwhile, 45 (100%) of the decreased abundance proteins were cytosolic proteins without a transmembrane region and/or single peptide. This study supported our previous finding (Ujang et al., (2018) [7]) in which there were mixed membrane and cytosolic proteins in the membrane fraction. We have confirmed and added value to the previous report that several increased abundance cytosolic proteins are present in the *E. histolytica* membrane fraction in this study.

There was a combination of membrane and cytosolic proteins among increased abundance proteins. Membrane proteins are 70 kDa HSP putative, 3-oxo 5-alpha-steroid 4-dehydrogenase, calcium-transporting ATPase, NAD(P) transhydrogenase, protein SEY1 homolog 2, cell surface proteases gp63 putative, Ehsyntaxin 1A (fragment) and Ehsyntaxin B. Furthermore, Rho family GTPase, calreticulin putative, 60S acidic ribosomal protein, uncharacterized proteins, and Rho and Rab family GTPase were cytosolic proteins (Table 2). For example, a well-studied protein that related to the parasite’s pathogenicity over past decades was calreticulin and this protein was found to be increased in abundance in the present study. Calreticulin is an immunogenic molecule involved in binding and cellular process, has no transmembrane domain and/or signal peptide. It is able to induce a host immune response. Furthermore, during the initial stage of the infection, increased expression of this parasite protein can be seen [8,9]. 

In this study, type A flavoprotein (EHI_152650), an oxidoreductase enzyme, was found to be decreased in abundance together with other stress response enzymes; superoxide dismutase (EHI_159160), peptidylprolyl isomerase (EHI_125840), and heat shock protein 70 putative (EHI_052860) in the membrane fraction. An interesting observation by Macfarlane and Singh (2005) showed the stress response in the nonvirulent *E. histolytica* was affected if a group of genes decreased in their expression, for instance, type A flavoprotein, which plays a role in the detoxification of oxygen and nitric oxide [8].

Different isoforms of Rho family GTPase and 70 kDa heat shock proteins were found to be increased and decreased in abundance in this study (Table 2 and Table 3). The Rho family GTPase protein (EHI_129750) and 70 kDa heat shock protein putative (EHI_199590) were increased in abundance, while its isoforms Rho family GTPase (EHI_192450 and EHI_146180) and heat shock protein 70 putative (EHI_052860) were decreased in abundance by comparing the two fractions. The 70 kDa heat shock protein and Rho family GTPase family proteins play important roles in the virulence of the parasite together with other identified proteins, for example, Gal/GalNAc subunit, NAD(P) transhydrogenase alpha and calreticulin [9]. From the functional classification analysis, Rho family GTPase (EHI_129750) is involved in catalytic activity and binding and increased abundance of GTPase proteins may give a possibility of effective colonization and invasion of the trophozoites in the host [10]. Soid-Raggi et al., (1998) reported that the G protein that the Rho family GTPase belongs has the possible elements of signal transduction in the trophozoite’s interaction with fibronectin [10]. Meanwhile, increased abundance of 70 kDa heat shock protein will protect amoeba because the heat shock protein (HSP) will be produced if an immediate temperature spike occurs [11].

Amoeba also contains peroxiredoxin (Prx), superoxide dismutase (EHI_159160), flavoprotein A (EHI_096710), ferredoxin (EHI_051060), thioredoxin (EHI_133970) and thioredoxin reductase for its protection against oxidative stress. Furthermore, the pathogenesis of *Entamoeba* has a strong association with antioxidative defense mechanisms. In this study, only the thioredoxin enzyme, which is known as a sensitive buffer was found to be increased in abundance [12]. Meanwhile, superoxide dismutase, flavoprotein A and ferredoxin were found to be decreased in abundance. Superoxide dismutase plays an important role in demolishing the cell radical of superoxide anions, which harms the *E. histolytica* biological system.

Another cytosolic protein, F-actin-capping protein subunit beta (EHI_005020), is a significant protein that has been detected because it takes roles in many functions including binding activities (molecular function); cellular process, biogenesis, and biological regulation (biological process); and cell/cell part (cellular component), which can inhibit the elongation of actin filaments to assure the parasite is motile [13].

Highly significant interactions (*p* < 3.66e × 10^−11^) were predicted in decreased abundance proteins when compared to increased abundance proteins (*p* < 1.91 × 10^−7^) (Figure 4A,B). This result is because all of the decreased abundance proteins are cytosolic proteins, which have been extensively studied previously. The results showed a few membrane and cytosolic proteins in the membrane fraction were predicted to be associated by the protein–protein interaction analysis.

Interesting interactions included calreticulin (EHI_136160) as a central protein, which was linked to two membrane proteins and three cytosolic proteins (Figure 4A). Calreticulin, a cytosolic protein, interacted with two membrane proteins, 70 kDa HSP (EHI_199590) and protein disulfide isomerase (EHI_071590) (Figure 4A). EhPDI (protein disulfide isomerase) belongs to the PDI family, which is known as ER’s lumen soluble marker. There was a report by Salgado et al., (2005) where a nuclear fraction of the amoeba was associated with the lack of endoplasmic reticulum (ER) markers. Calreticulin is an ER protein specifically located at the membrane of the ER periphery [14] and it promotes sites for glycosylation and calcium-binding. Overexpression of this protein in patients with amoebic liver abscess was associated with an immunogenic response at the initial stage of infection [15]. Both PDI and calreticulin associated with ER, which explains the possibility of the interaction occuring between these proteins in this study. On the other hand, calreticulin linked to other proteins such as 70 kDa HSP, actin, thioredoxin and elongation factor 1 due to these proteins being involved in similar molecular functions and biological processes categories. For instance, both 70 kDa HSP and calreticulin were involved in binding and cellular activity.

## 5. Conclusions

In conclusion, this study has confirmed the presence of mixed differential abundance proteins in membrane versus cytosolic fractions. Furthermore, the functional analysis and protein–protein interactions among these proteins were predicted. Several membrane and cytosolic proteins that showed significant interactions merit further investigation to confirm their localization and function on the membrane part to further our understanding of the *E. histolytica* pathogenesis.

## Figures and Tables

**Table 1:** Increased and decreased abundance proteins in the membrane versus cytosolic fractions (q < 0.05) by three different methods.

Extraction Method	Increased	Decreased
	Fc > 2-Fold; q < 0.05	Fc > 2-Fold; q < 0.05
Conventional Method	19	23
ProteoExtract Kit	4	2
ProteoPrep Kit	31	20
TotalL	54	45

**Table 2:** Increased abundant membrane and cytosolic proteins in the membrane versus cytosolic fractions (q < 0.05).

TM and/or Signal Peptide	Fold Change	Mapped ID	Gene Name
**-**	2.0141	EHI_078310	Uncharacterized protein
-	2.3102	EHI_129750	Rho family GTPase
-	2.0333	EHI_198580	Uncharacterized protein
Yes	2.4443	EHI_165070	Estradiol 17-beta-dehydrogenase putative
-	2.6115	EHI_023360	Alpha-amylase family protein
-	3.6739	EHI_065790	Rab family GTPase (Fragment)
-	2.3510	EHI_152280	Serine palmitoyltransferase putative
Yes	2.6371	EHI_199590	70 kDa heat shock protein putative
-	2.2277	EHI_120360	Grainin putative
-	2.1083	EHI_006980	Gal galnac lectin subunit igl1
-	2.5152	EHI_102170	Elongation factor 1-alpha
Yes	2.0661	EHI_024350	Uncharacterized protein
-	2.0284	EHI_107290	Actin
-	2.6989	EHI_047800	Uncharacterized protein
Yes	2.5246	EHI_076870	3-oxo 5 alpha-steroid 4-dehydrogenase domain-containing
Yes	2.9713	EHI_054830	Calcium-transporting ATPase
-	4.4102	EHI_050520	Uncharacterized protein
-	2.8741	EHI_169280	EhRab7E protein
Yes	3.3411	EHI_014030	NAD(P) transhydrogenase subunit alpha, putative
Yes	2.6606	EHI_025100	G protein-coupled receptor 1
Yes	4.1994	EHI_012270	170 kDa surface lectin
Yes	2.3614	EHI_141330	Uncharacterized protein
Yes	2.5185	EHI_120590	Uncharacterized protein
-	2.0883	EHI_041950	Vacuolar protein sorting 35, putative
Yes	2.8047	EHI_164800	Cysteine protease binding protein family 1
-	3.411	EHI_136160	Calreticulin putative
Yes	3.1331	EHI_098510	Uncharacterized protein
-	4.4288	EHI_192810	small GTPase Rab7A
-	2.9434	EHI_147010	Long-chain-fatty-acid—CoA ligase, putative
Yes	2.3719	EHI_071590	Protein disulfide isomerase putative
-	2.2623	EHI_133970	Thioredoxin putative
Yes	2.8980	EHI_158240	3-ketoacyl-CoA synthase
Yes	2.2738	EHI_139030	EhSyntaxin 1A (Fragment)
-	2.3774	EHI_107250	small GTPase Rab11B
-	3.3584	EHI_140260	Copine putative
Yes	2.2996	EHI_054180	Protein SEY1 homolog 2
Yes	3.1250	EHI_200230	Cell surface protease gp63 putative
-	2.3851	EHI_127030	Uncharacterized protein
-	2.4601	EHI_007330	Beta-hexosaminidase
Yes	2.0039	EHI_067090	Uncharacterized protein
Yes	2.5342	EHI_178470	Cysteine protease binding protein family 6
Yes	3.1223	EHI_042370	Galactose-specific adhesin 170 kDa subunit, putative
Yes	2.7603	EHI_021410	EhSyntaxin B
Yes	3.8750	EHI_133900	Galactose-inhibitable lectin 170 kDa subunit putative
Yes	3.4012	EHI_148790	Gal galNac lectin light subunit
-	2.7452	EHI_175460	60S acidic ribosomal protein P0
Yes	2.4905	EHI_101230	P-glycoprotein 6
-	2.5152	EH_052400	Elongation factor-1-alpha

**Table 3:** Decreased abundance of cytosolic proteins in the membrane versus cytosolic fractions (q < 0.05). These proteins lack a transmembrane (TM) and signal peptide domain.

Mapped ID	Fold Change	Gene Name/Symbol	PANTHER (Family/Subfamily)
EHI_130700	2.953	Enolase putative	Enolase (Pthr11902:Sf1)
EHI_052860	2.9703	Heat shock protein 70 putative	Ribosome-Associated Molecular Chaperone Ssb1-Related (Pthr19375:Sf395)
EHI_104390	2.6725	Actin binding protein putative	-
EHI_042170	3.2691	Aminoacyl-histidine dipeptidase putative	Cytosol Non-Specific Dipeptidase (Pthr43501:Sf1)
EHI_048310	2.4029	EhSec24C	Secretory 24cd, Isoform C (Pthr13803:Sf38)
EHI_023110	3.1633	NADP-dependent Alcohol dehydrogenase	Dehydrogenase 1, Putative-Related (Pthr42813:Sf4)
EHI_125840	3.0822	Peptidyl-prolyl cis-trans isomerase	Peptidyl-Prolyl Cis-Trans Isomerase D-Related (Pthr11071:Sf380)
EHI_166810	3.7550/ 2.6014	Elongation factor 2	Elongation Factor 2 (Pthr42908:Sf10)
EHI_098570	3.1517	Fructose 1 6-bisphosphate aldolase putative	D-Tagatose-1,6-Bisphosphate Aldolase Subunit Gaty-Related (Pthr30304:Sf0)
EHI_155410	2.8691	40S ribosomal protein S11, putative	40s Ribosomal Protein S11 (Pthr10744:Sf9)
EHI_104630	2.5185	Filamin 2 putative	Zgc:100997 (Pthr19961:Sf58)
EHI_006670	2.5009	Uncharacterized protein	Expressed Protein (Pthr19308:Sf14)
EHI_198930	3.7526	Actin putative	Actin-Related Protein 3b (Pthr11937:Sf31)
EHI_021360	2.5234	Uncharacterized protein	Translationally Controlled Tumor Protein (Pthr11991:Sf0)
EHI_192450	2.0262	Rho family GTPase	Ras-Related Protein Rac1-Related (Pthr24072:Sf281)
EHI_146180	2.3813	Rho family GTPase	Ras-Related Protein Rac1-Related (Pthr24072:Sf281)
EHI_104560	2.8300	Cortexillin putative	Cortexillin-2 (Pthr23167:Sf67)
EHI_176140	2.7438/ 2.4697	Profilin	Profilin (Pthr11604:Sf0)
EHI_174180	2.1882	Skp1 family protein	S-Phase Kinase-Associated Protein 1 (Pthr11165:Sf24)
EHI_165350	2.6236	Malate dehydrogenase putative	Hydroxycarboxylate Dehydrogenase B-Related (Pthr11091:Sf0)
EHI_159160	2.4921	Superoxide dismutase	Superoxide Dismutase [Fe] 2, Chloroplastic (Pthr42769:Sf3)
EHI_011270	2.2522	Uncharacterized protein	Si:Ch211-282j17.12-Related (Pthr18884:Sf83)
EHI_178960	2.7608/ 2.5979	Acetyl-CoA synthetase, putative	Acetate--Coa Ligase [Adp-Forming] (Pthr43334:Sf1)
EHI_152680	2.1693	EH-domain containing protein putative	At21416p (Pthr11216:Sf31)
EHI_147570	2.1748/ 2.7106	Rho GDP exchange inhibitor, putative	Ld16419p (Pthr10980:Sf3)
EHI_193330	2.2123	60S ribosomal protein L23 putative	60s Ribosomal Protein L23 (Pthr11761:Sf8)
EHI_110180	2.4310	Myosin heavy chain	Myosin Heavy Chain, Non-Muscle (Pthr45615:Sf40)
EHI_051060	3.7812	Pyruvate ferredoxin oxidoreductase	Pyruvate-Flavodoxin Oxidoreductase-Related (Pthr32154:Sf0)
EHI_125950	3.1278	Alcohol dehydrogenase putative	Alcohol Dehydrogenase Yqhd (Pthr43633:Sf1)
EHI_006810	2.7083	14-3-3 protein 3	14-3-3 Protein Zeta (Pthr18860:Sf106)
EHI_152650	2.5095	Type A flavoprotein, putative	Diflavin Flavoprotein A 2-Related (Pthr32145:Sf11)
EHI_044970	3.9670	Malic enzyme	Nadp-Dependent Malic Enzyme (Pthr43237:Sf4)
EHI_091250	2.8148	Arp2/3 complex 34 kDa subunit	Actin-Related Protein 2/3 Complex Subunit 2 (Pthr12058:Sf0)
EHI_005020	2.5429	F-actin-capping protein subunit beta	F-Actin-Capping Protein Subunit Beta (Pthr10619:Sf0)
EHI_009530	2.136	Pyruvate, phosphate dikinase	Pyruvate, Phosphate Dikinase 1, Chloroplastic (Pthr22931:Sf9)

**Table 4:** Line colors explanation.

Color	Evidence
Red	Presence of fusion
Green	Neighborhood
Blue	Cooccurrence
Purple	Experimental
Yellow	Text mining
Light blue	Database
Black	Coexpression

**Note:** Different color lines indicate types of interaction evidence used in predicting the association.

## Data Availability

Not applicable.

## Supplementary Materials

The following are available online at https://www.mdpi.com/article/10.3390/membranes11060376/s1, Supplementary file 1. Table S1. Classification of Increased Abundance Proteins by PANTHER DB. Table S2. Classification of decreased abundance proteins by PANTHER DB.
