# Supplementary material for: Entamoeba histolytica: Proteomics Bioinformatics Reveal Predictive Functions and Protein–Protein Interactions of Differentially Abundant Membrane and Cytosolic Proteins
Source: Membranes (Basel). 2021 May 21;11(6):376. doi: 10.3390/membranes11060376 (PMC8224062; doi:10.3390/membranes11060376)
Supplement: Supplementary file 1 [file membranes-11-00376-s001.zip › membranes-1183562-supplementary.pdf]

membranes

Supplementary Materials:

Entamoeba histolytica: Proteomics bioinformatics revealed predictive functions and protein- protein interactions of differentially abundant membrane and cytosolic protein

Norhidayah Azmi [1] and Nurulhasanah Othman [1,*]

[1] Institute for Research in Molecular Medicine (INFORMM), Universiti Sains Malaysia, 11800, Pulau Pinang, Malaysia; hidayah84@student.usm.my

* Correspondence: nurulhasanah@usm.my

Citation: Azmi, N.; Othman, N. *Entamoeba histolytica*: Proteomics Bioinformatics Reveal Predictive Functions and Protein-Protein Interactions of Differentially Abundant Membrane and Cytosolic Proteins. **2021**, *11*, 376. https://doi.org/10.3390/membranes11060376

Academic Editor: Igor Zhukov

Received: 30 March 2021
Accepted: 18 May 2021
Published: 21 May 2021

Publisher's Note: MDPI stays neutral with regard to jurisdictional claims in published maps and institutional affiliations.

Copyright: © 2021 by the authors. Licensee MDPI, Basel, Switzerland. This article is an open access article distributed under the terms and conditions of the Creative Commons Attribution (CC BY) license (http://creativecommons.org/licenses/by/4.0/).

Table S1. Classification of Increased Abundance Proteins by PANTHER DB (Membrane proteins are bolded)

Accession Number	Gene Name	PANTHER (Family/Subfamily)
Catalytic Activity (Molecular Function)		
EHI_129750	Rho family GTPase	Ras-related protein rac1-related (pthr24072: sf281)
**EHI_199590**	**70 kDa heat shock protein putative**	**Endoplasmic reticulum chaperone bip (pthr19375: sf144)**
**EHI_076870**	**3-oxo 5 alpha-steroid 4-dehydrogenase domain**	**Sc2 (pthr10556: sf28)**
**EHI_054830**	**Calcium-transporting ATPase**	**Calcium-transporting ATPase (pthr24093: sf369)**
**EHI_014030**	**NAD(P) transhydrogenase subunit alpha, putative**	**Nicotinamide nucleotide transhydrogenase (pthr10160: sf19)**
**EHI_054180**	**Protein SEY1 homolog 2**	**Protein sey1 (pthr45923: sf2)**
EHI_200230	Cell surface protease gp63 putative	Leishmanolysin-like peptidase (pthr10942: sf0)
Binding (Molecular Function)		
EHI_129750	Rho family GTPase	Ras-related protein rac1-related (pthr24072: sf281)
**EHI_199590**	**70 kDa heat shock protein putative**	**Endoplasmic reticulum chaperone bip (pthr19375: sf144)**
**EHI_014030**	**NAD(P) transhydrogenase subunit alpha, putative**	**Nicotinamide nucleotide transhydrogenase (pthr10160: sf19)**
EHI_136160	Calreticulin putative	Calreticulin (pthr11073: sf2)
**EHI_139030**	**EhSyntaxin 1A (Fragment)**	**Syntaxin-1a (pthr19957: sf41)**
**EHI_021410**	**EhSyntaxin B**	**Ld23667p (pthr19957: sf38)**
EHI_175460	60S acidic ribosomal protein	60s acidic ribosomal protein p0 (pthr45699: sf3)
**Accession Number**	**Gene Name**	**PANTHER (Family/Subfamily)**
Cellular Process (Biological Process)		
EHI_078310	Uncharacterized protein	Protein yippee-like (pthr14255: sf3)
EHI_129750	Rho family GTPase	Ras-related protein rac1-related (pthr24072: sf281)
EHI_065790	Rab family GTPase	Ld44762p (pthr47980: sf24)

**EHI_199590**	**70 kDa heat shock protein putative**	**Endoplasmic reticulum chaperone bip (pthr19375: sf144)**
**EHI_076870**	**3-oxo 5 alpha-steroid 4-dehydrogenase domain-contai**	**Sc2 (pthr10556: sf28)**
**EHI_014030**	**NAD(P) transhydrogenase subunit alpha, putative**	**Nicotinamide nucleotide transhydrogenase (pthr10160: sf19)**
EHI_136160	Calreticulin putative	Calreticulin (pthr11073: sf2)
**EHI_139030**	**EhSyntaxin 1A (Fragment)**	**Syntaxin-1a (pthr19957: sf41)**
**EHI_054180**	**Protein SEY1 homolog 2**	**Protein sey1 (pthr45923: sf2)**
**EHI_021410**	**EhSyntaxin B**	**Ld23667p (pthr19957: sf38)**
EHI_175460	60S acidic ribosomal protein	60s acidic ribosomal protein p0 (pthr45699: sf3)
Cell/Cell Part (Cellular Component)		
EHI_078310	Uncharacterized protein	Protein yippee-like (pthr14255: sf3)
EHI_129750	Rho family GTPase	Ras-related protein rac1-related (pthr24072: sf281)
EHI_065790	Rab family GTPase (Fragment)	Ld44762p (pthr47980: sf24)
**EHI_199590**	**70 kDa heat shock protein putative**	**Endoplasmic reticulum chaperone bip (pthr19375: sf144)**
**EHI_076870**	**3-oxo 5 alpha-steroid 4-dehydrogenase domain-contai**	**Sc2 (pthr10556: sf28)**
**EHI_014030**	**NAD(P) transhydrogenase subunit alpha, putative**	**Nicotinamide nucleotide transhydrogenase (pthr10160: sf19)**
EHI_136160	Calreticulin putative	Calreticulin (pthr11073: sf2)
**EHI_139030**	**EhSyntaxin 1A (Fragment)**	**Syntaxin-1a (pthr19957: sf41)**
**EHI_054180**	**Protein SEY1 homolog 2**	**Protein sey1 (pthr45923: sf2)**

Table S2. Classification of decreased abundance proteins by PANTHER DB.

Mapped ID	Gene Name/ Symbol	PANTHER (Family/Subfamily)
**Binding / Cellular Process / Cell Part**		
EHI_052860	Heat shock protein 70 putative	Ribosome-associated molecular chaperone ssb1-related (pthr19375: sf395)
EHI_125840	Peptidyl-prolyl cis-trans isomerase	Peptidyl-prolyl cis-trans isomerase d-related (pthr1107: sf380)
EHI_166810	Elongation factor 2	Elongation factor 2 (pthr42908: sf10)
EHI_104630	Filamin 2 putative	Zgc:100997 (pthr19961: sf58)
EHI_198930	Actin putative	Actin-related protein 3b (pthr11937: sf31)
EHI_192450	Rho family GTPase	Ras-related protein rac1-related (pthr24072: sf281)
EHI_146180	Rho family GTPase	Ras-related protein rac1-related (pthr24072: sf281)
EHI_176140	Profilin	Profilin (pthr11604: sf0)
EHI_174180	Skp1 family protein	S-phase kinase-associated protein 1 (pthr11165: sf24)
EHI_147570	Rho GDP exchange inhibitor, putative	Ld16419p (pthr10980: sf3)
EHI_091250	Arp2/3 complex 34 kDa subunit	Actin-related protein 2/3 complex subunit 2 (pthr12058: sf0)
EHI_005020	F-actin-capping protein subunit beta	F-actin-capping protein subunit beta (pthr10619: sf0)
